# Complete Genome Sequence of a Newcastle Disease Virus Isolated from a Rock Dove (Columba livia) in the Russian Federation

**DOI:** 10.1128/genomeA.01514-14

**Published:** 2015-01-29

**Authors:** Kseniya S. Yurchenko, Mariya V. Sivay, Alexandra V. Glushchenko, Sergey V. Alkhovsky, Alexey M. Shchetinin, Michail Y. Shchelkanov, Alexander M. Shestopalov

**Affiliations:** a FSBI Scientific Center of Clinical and Experimental Medicine, Siberian Branch of the Russian Academy of Medical Sciences, Novosibirsk, Russian Federation; b D.I. Ivanovsky Institute of Virology, Russian Academy of Medical Sciences, Moscow, Russian Federation

## Abstract

We report here the complete genome sequence of a Newcastle disease virus (NDV) isolate, NDV/Altai/pigeon/770/2011, isolated from a rock dove in the Russian Federation. On the basis of phylogenetic analysis, this strain was clustered into genotype VIb class II.

## GENOME ANNOUNCEMENT

Newcastle disease virus (NDV), or avian paramyxovirus type-1 (APMV-1), causes highly infectious and economically significant disease affecting birds of various species worldwide (1). NDV belongs to the genus *Avulavirus* of the family *Paramyxoviridae* (2) and possesses a single-stranded, nonsegmented, and negative-sense RNA. An ND-like disease is also found in pigeons. This variant of the virus is called pigeon paramyxovirus type -1 (PPMV-1), and this isolate is an antigenic variant of NDV (3).

We report here the complete genome sequence of the NDV/Altai/pigeon/770/2011 strain, isolated from a rock dove (Columba livia) in the Russian Federation (Altai Region) in fall 2011. The virus was propagated in the allantoic cavity of 9-day-old embryonated hen eggs, from nonvaccinated chickens. Molecular, biological, and phylogenetic analyses of NDV/Altai/pigeon/770/2011 were conducted.

NDVs have been categorized into velogenic (highly virulent), mesogenic (intermediately virulent), and lentogenic (nonvirulent) pathotypes. This classification was determined according to standard pathogenicity tests based on the level of viral toxicity to embryonated eggs and chickens (1, 4). NDV/Altai/pigeon/770/2011 was classified as mesogenic NDV with a mean death time (MDT) of 76 h and with an intracerebral pathogenicity index (ICPI) of 0.68.

The other commonly used classification is based on the fusion protein sequence. The F protein cleavage site is a major determinant of NDV pathogenicity. The cleavage sites of virulent NDV strains usually contain multiple basic residues, whereas avirulent strains have fewer basic residues (1). However, PPMV-1 strains with a motif associated with high virulence can show reduced virulence, or even no virulence, in chickens but cause morbidity in pigeons (5).

Viral RNA was isolated using TRIzolLS (Invitrogen, USA). cDNA was synthesized using the Random Hexamer primer with RevertAid Premium (Thermo Scientific, USA). The second DNA chain was synthesized using the NEBNext mRNA Second Strand Synthesis Module (NEB, USA) following the manufacturer’s instructions. DNA libraries were made by TruSeq DNA Sample Prep Kits version 2 (Illumina, USA) according to the manufacturer’s instructions. DNA libraries were sequenced by MiSeq (Illumina, USA) using MiSeq Reagent Kits version 2 (300PE) according to the manufacturer’s instructions. Bioinformatic analysis was conducted using CLC Genomics Workbench version 5.5 software (CLC bio, USA).

The complete genome of NDV/Altai/pigeon/770/2011 was sequenced and found to be 15,189 nt in length. It has six genes in the order 3′-N-P-M-F-HN-L-5′. The fusion protein cleavage site sequence contains multiple basic amino acids 112K-R-Q-K-R116 at the C terminus of the F2 protein and phenylalanine at position 117, the N terminus of the F1 protein. According to the motif at the F2/F1 cleavage site, NDV/Altai/pigeon/770/2011 associates with the virulent pathotype. However, biological characteristics confirm that the virus is mesogenic in chickens. A phylogenetic tree showed that NDV/Altai/pigeon/770/2011 was classified into genotype VIb class II, a group of viruses that caused panzootic infections among pigeons in the 1980s (6).

Phylogenetic analysis showed that NDV/Altai/pigeon/770/2011 clades with other pigeon NDV viruses and is related to viruses isolated in the Russian Federation in 2005 and 2009.

### Nucleotide sequence accession number.

The complete genome sequence of NDV/Altai/pigeon/770/2011 has been deposited in GenBank under the accession number KJ920204.
